# Variant effect predictions capture some aspects of deep mutational scanning experiments

**DOI:** 10.1186/s12859-020-3439-4

**Published:** 2020-03-17

**Authors:** Jonas Reeb, Theresa Wirth, Burkhard Rost

**Affiliations:** 1 Department of Informatics, Bioinformatics & Computational Biology - i12, TUM (Technical University of Munich), Boltzmannstr 3, 85748 Garching/Munich, Germany; 2 Institute for Advanced Study (TUM-IAS), Lichtenbergstr 2a, 85748 Garching/Munich, Germany; 3 TUM School of Life Sciences Weihenstephan (WZW), Alte Akademie 8, Freising, Germany; 4 Department of Biochemistry and Molecular Biophysics, Columbia University, 701 West, 168th Street, New York, NY 10032 USA

**Keywords:** Sequence variation, Variant effect prediction, Deep mutational scanning, Non-synonymous sequence variant, Missense variant, Single nucleotide variant

## Abstract

**Background:**

Deep mutational scanning (DMS) studies exploit the mutational landscape of sequence variation by systematically and comprehensively assaying the effect of single amino acid variants (SAVs; also referred to as missense mutations, or non-synonymous Single Nucleotide Variants – missense SNVs or nsSNVs) for particular proteins. We assembled SAV annotations from 22 different DMS experiments and normalized the effect scores to evaluate variant effect prediction methods. Three trained on traditional variant effect data (PolyPhen-2, SIFT, SNAP2), a regression method optimized on DMS data (Envision), and a naïve prediction using conservation information from homologs.

**Results:**

On a set of 32,981 SAVs, all methods captured some aspects of the experimental effect scores, albeit not the same. Traditional methods such as SNAP2 correlated slightly more with measurements and better classified binary states (effect or neutral). Envision appeared to better estimate the precise degree of effect. Most surprising was that the simple naïve conservation approach using PSI-BLAST in many cases outperformed other methods. All methods captured beneficial effects (gain-of-function) significantly worse than deleterious (loss-of-function). For the few proteins with multiple independent experimental measurements, experiments differed substantially, but agreed more with each other than with predictions.

**Conclusions:**

DMS provides a new powerful experimental means of understanding the dynamics of the protein sequence space. As always, promising new beginnings have to overcome challenges. While our results demonstrated that DMS will be crucial to improve variant effect prediction methods, data diversity hindered simplification and generalization.

## Background

Recent human sequencing projects conclude that we all carry about 10,000 single amino acid variants (**SAVs**; also referred to as missense mutations, or non-synonymous Single Nucleotide Variants: **nsSNVs**) with respect to the “reference genome” and by 20,000 for every pair of unrelated individuals [1, 2]. Many of these SAVs are assumed to be neutral, while others might change protein function, contributing to complex phenotypes and causing diseases. Unfortunately, the gap between SAVs with and without experimental characterization continues to widen [3]: for only one in 10,000 of the known SAVs some experimental information is available [4, 5]. On top, many of those for which something is known may be incorrect disease associations [6]. Without improving the ability to interpret SAV effects, both on the level of the organism and the protein, the promise of precision medicine will remain, importantly unmet [7–10].

Through the increased efficiency of sequencing, a procedure formerly used primarily in silico [11, 12] has become feasible for experiments, namely assessing the effect of all possible SAVs in a protein, i.e. all possible amino acid mutations. In such deep mutational scanning (**DMS**) studies [13, 14], a sequence library with all possible variants is subjected to selection. In the simplest case, the (logarithmic) difference between sequence frequencies with and without selection pressure yield an effect score for individual or combinations of variants [8, 15–17]. Variants with beneficial and deleterious effect on protein function are discovered together with a quantification of how much effect. Thus, DMS aims at measuring the landscape of functional fitness for select proteins [18].

DMS also screens proteins for improved drug binding, antibody affinity, using non-native chemical stresses, or non-proteinogenic amino acids, and on synthetic proteins [19–26]. Finally, DMS share objectives with directed evolution, benefiting protein engineering [14].

One major challenge for DMS is the development of an assay to measure effect. Evaluating proteins with multiple functions requires multiple assays [8]. For instance, for the Ubiquitin-60S ribosomal protein L40 variant effects have been assessed through their direct impact on yeast growth and through the impaired activation by the E1 enzyme [27, 28]. Similarly, BRCA1 has been assayed through E3 ubiquitin ligase activity and through BARD1 binding and transcript abundance [29, 30]. Even for the same assay, specific experimental conditions might influence measurements [31]. Recently, a protocol for measuring protein abundance has been suggested as a proxy for function and applicable to many proteins [32]. The conclusions from DMS studies are limited by the validity of their functional assays; inferences of more complex effect relationships such as disease risk or clinically actionable pathogenicity often remain too speculative [8, 17]. On top, variants might affect molecular function as assayed by DMS although being clinically benign, i.e. not causing disease.

Long before experimental DMS, prediction methods had addressed the same task in silico [33–41]. These methods were developed on very limited data; many focused on disease-causing SAVs from OMIM [42], others used databases such as PMD [43] cataloguing variants by effect upon protein function or structure. CADD solved the problems of data limitation and bias by considering all mutations that have become fixed in the human population as neutral and a simulated set of all other variants as having an effect [35]. The training dataset determines the type of effect methods can learn. Consequently, methods differ and work only on the type of SAV used for development. Given the limitations in today’s data, all methods have been optimized on relatively small, unrepresentative subsets: fewer than 85,000 of all possible 217 million human SAVs (< 0.04%) have some experimental annotations [44, 45]. Methods agree much more with each other for SAVs with than for those without annotations [46].

DMS datasets constitute a uniquely valuable resource for the evaluation of current SAV effect prediction methods [17, 47, 48], not the least, because most have not used those data. The Fowler lab has, recently, published an excellent analysis of prediction methods on DMS datasets and developed a new regression-based prediction method, Envision, trained only on DMS data [49]. Here, we focus on the analysis of a larger set of DMS studies and present trends in their correlation with SAV effects predicted by four variant effect prediction methods.

## Results

### DMS studies not complete yet

Our Deep Mutational Scanning (DMS) analyses began with 22 separate experimental datasets from 18 unique proteins, since some experiments were performed on the same protein (Supplementary Online Material (SOM), Fig. S1a, Table S1) [29, 30, 32, 50–65]. In total the set contained 68,447 variants (Fig. S1); 2358 (3%) of these were synonymous, the other 97% constituted SAVs (or missense mutations).

Only ten of the 22 sets (45%) scored some variants for at least 98% of the residues (Table S1). Four DMS studies provided functional scores for over 90% of all possible 19 non-native SAVs. On average, 66% of the residues had SAVs with both deleterious and beneficial effects (Table S2; those two could be seen as “disruptive variants” arching over gain- and loss-of-function). Most SAVs were beneficial for only 3 of 22 studies (14%), for the other 19 studies deleterious outnumbered beneficial SAVs by factors of 1.5–22.5 (Fig. S1b). Due to asymmetries in numbers and experimental fidelity, deleterious and beneficial SAVs were analyzed separately.

### Some correlation achieved by all methods

**Fig. 1:**
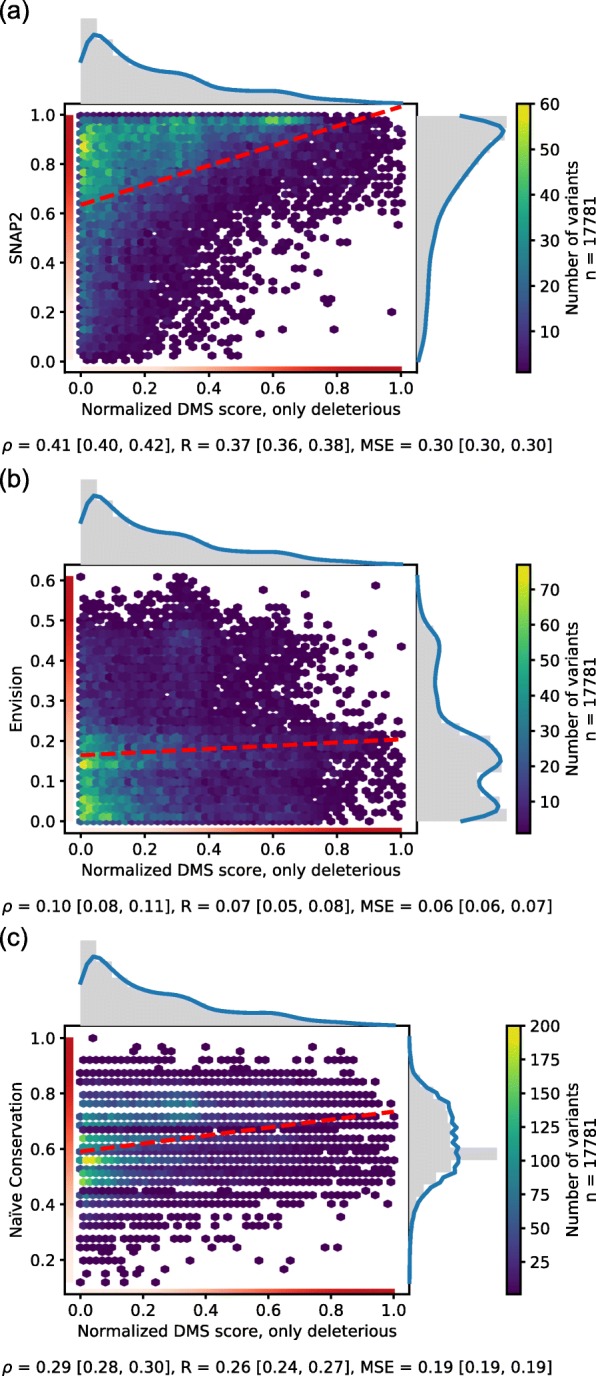
DMS experiments vs. variant effect predictions. In a hexbin plot, 17,781 deleterious effect SAVs in *SetCommon* were compared to normalized scores for three prediction methods (SNAP2 [38], Envision [49], and Naïve Conservation). Values on both axes range from 0 (neutral) to 1 (maximal effect) as denoted by the gradient from white (neutral) to red (effect). Dashed red lines give linear least-squared regressions. Marginals denote distributions of experimental and predicted scores with a kernel density estimation overlaid in blue. The footer denotes Spearman ρ, Pearson R and the mean squared error together with the respective 95% confidence intervals. The method scores are given on the y-axes and reveal the method: **a** SNAP2, **b** Envision – the only method trained on DMS data, **c** Naïve Conservation read off PSI-BLAST profiles

*SetCommon* constituted a subset of all 22 datasets with 32,981 effect SAVs (17,781 deleterious) for which we had predictions from each method (Table 1). Although all predictions differed from the experiments, all correlated slightly positively for deleterious SAVs (Spearman ρ ≥ 0.1, Fig. 1a-c, Tables 2, S3). The 95% confidence intervals (CIs) of methods did not overlap, and their differences were statistically significant (Table S4).

**Table 1:** Number of SAVs in aggregated datasets^a^

	Number of SAVs			
	Total	Neutral	Deleterious	Beneficial
SetAll	66,089	818 ^b^	45,382	19,889
SetCommon	32,981	0	17,781	15,200
SetCommonSyn90	15,621	8926	4545	2150
SetCommonSyn95	15,621	10,587	3209	1825
SetCommonSyn99	15,621	13,506	1548	567

^a^*SetAll* depicts the total number of SAVs collected, while *SetCommon* contains only SAVs with predictions from every analyzed method. *SetCommonSyn* contains all SAVs with predictions where a thresholding scheme could be applied to yield classification of SAVs into neutral and effect (see Methods). The number of SAVs in every single DMS experiment are depicted in Fig. S1 and Table S1

^b^The ccdB set classifies variant effect in categories and contains 818 non-synonymous variants which fall in the same category as the wild-type. Hence these SAVs could be considered neutral

**Table 2:** Pearson ρ and mean squared error (MSE) for methods on *SetCommon*^a^

	deleterious SAVs (*n* = 17,781)		beneficial SAVs (*n* = 15,200)	
	ρ	MSE	ρ	MSE
SNAP2	0.41 [0.40, 0.42]	0.3 [0.30, 0.30]	0.02 [0.01, 0.04]	0.23 [0.23, 0.24]
Envision	0.1 [0.08, 0.11]	0.06 [0.06, 0.07]	−0.14 [−0.16, − 0.13]	0.05 [0.04, 0.05]
Naïve Conservation	0.29 [0.27, 0.30]	0.19 [0.19, 0.19]	−0.08 [− 0.09, − 0.06]	0.19 [0.19, 0.20]

^a^*SetCommon* denotes the set of SAVs with predictions from every method (see Methods). ρ denotes Spearman ρ (higher is better), MSE the mean squared error (lower is better, Methods, SOM_Note3). Values in brackets are 95% confidence intervals

Both SIFT [39] and PolyPhen-2 [37] are optimized for capturing binary effects, not correlations, as confirmed by recent studies [47, 49]. Consequently, analysis for these was confined to binary predictions. SNAP2 [38] and Envision [49] scores appeared, overall, less binary (Figs. 1a-b). SNAP2 distributions were skewed toward high effect, while Envision also succeeded in detecting SAVs with less pronounced effects (Fig. 1a-b). Predictions by Naïve Conservation, based on PSI-BLAST profiles, correlated more with the DMS experiments than Envision (Fig. 1c).

### Envision might approximate experimental values best

When evaluating methods by the numerical difference between experimental and predicted variant effect scores (mean squared error, MSE), Envision appeared best, followed at considerable distance by Naïve Conservation and SNAP2 (Fig. 1, Table 2). However, its low MSE partially originated from predicting no SAV with strong effect (the highest Envision score was 61% of the possible maximum – 0.61). This resembled the experimental distribution skewed towards low effect (Fig. 1b, gray distributions next to x- and y-axes). Indeed, shuffling the prediction scores yielded the same MSE (Fig. S2a). Predicting a normal distribution around the experimental mean, performed slightly worse but still better than all other prediction methods (Fig. S2b). When considering each DMS measurement separately, Envision also appeared to perform best except for the transcriptional coactivator YAP1 (YAP1) with the most uniform distribution of effect scores (similar number of lowest, medium, and strongest effects observed; Fig. S3b, Table S5).

### All classification methods detect increasing effect strength

**Fig. 2:**
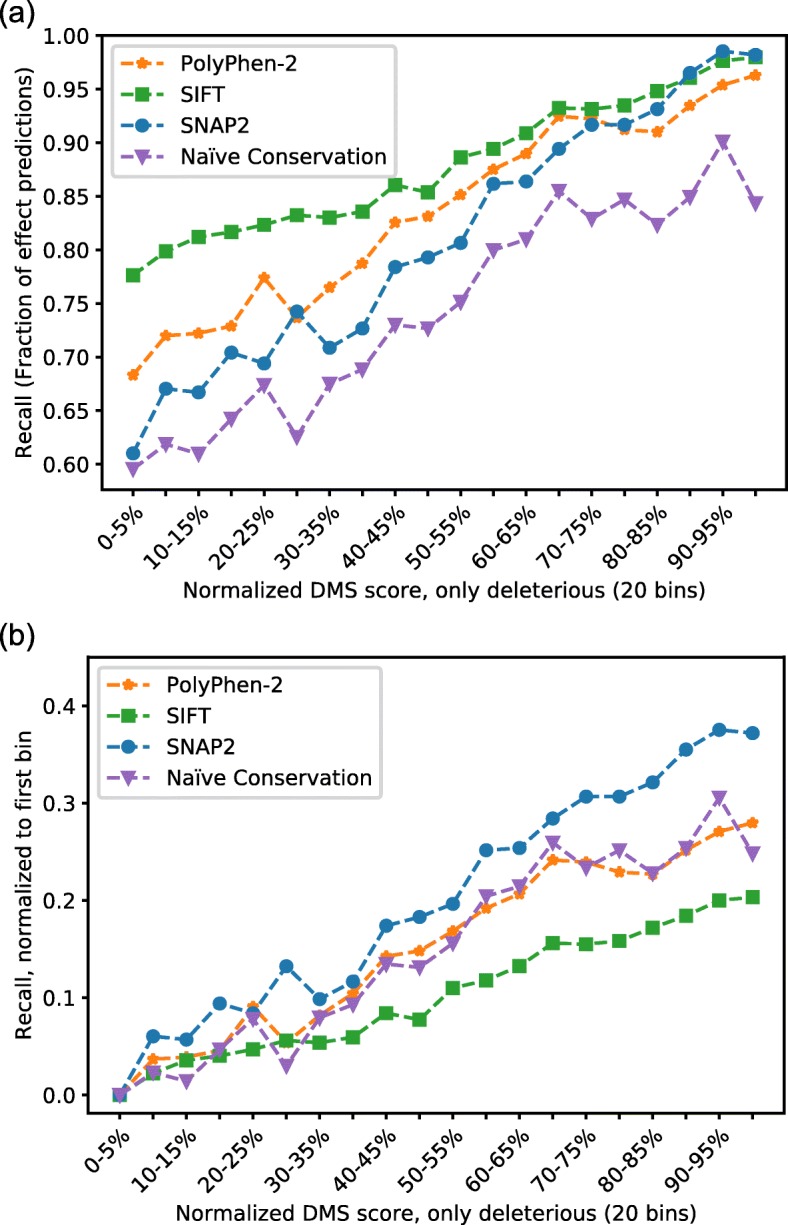
Recall proportional to deleterious DMS effect scores. The continuous normalized DMS scores with deleterious effect in *SetCommon* were split into 20 bins of equal size. **a** In each bin the fraction of SAVs predicted as having an effect by the binary classification methods (PolyPhen-2 [37], SIFT [39] and SNAP2 [38]) was shown. Naïve Conservation read off PSI-BLAST profiles was treated as an effect prediction when scores were above 0. For all other methods the default score thresholds were applied. **b** shows the values adjusted for the amount of effect predicted in the first bin

Do methods work better for SAVs with stronger observed effect? Toward this end, the experimental scores were sorted into 20 bins of increasing effect strength, and the effect predictions in each bin (here referred to as recall) were monitored for all prediction methods. All classification methods tended to reach higher recall levels for SAVs with stronger effects (Fig. 2a, higher values toward the right). Furthermore, all methods also show an increase without a clear saturation point showing that the range of increasing effect strength is detected. For some methods the difference between the least- and most-effect bins was higher than for others, i.e. their predictions distinguished more between high and low experimental scores (Fig. 2b).

### Beneficial effects difficult to predict

Unlike for deleterious SAVs, no method correlated, on average, with beneficial effect SAVs (− 0.14 ≤ ρ ≤ 0.02, Tables 2, S6, Fig. S4). Furthermore, most methods essentially predicted similar numbers or lower numbers of effect variants irrespective of the observed effect strength with the exception of SNAP2 that detected some high effect SAVs (Fig. S5). The conservation-based prediction also decreased substantially from a Spearman ρ of 0.29 for deleterious to − 0.08 for beneficial SAVs (Table 2, Fig. S4c). SNAP2 scores were shifted more toward lower effect than for deleterious SAVs (Fig. 1a and Fig. S4a, gray distributions). In contrast to Spearman ρ, the MSE for beneficial effect SAVs was similar to that for deleterious SAVs. Envision again was by far best (MSE = 0.05, Tables 2, S7, Fig. S6). However, although Envision used 25% beneficial effect SAVs for development (SOM_Note1), the correlation was much lower for beneficial than for deleterious SAVs (ρ = − 0.14 versus 0.1).

### Experimental agreement sets the benchmark for prediction methods

**Fig. 3:**
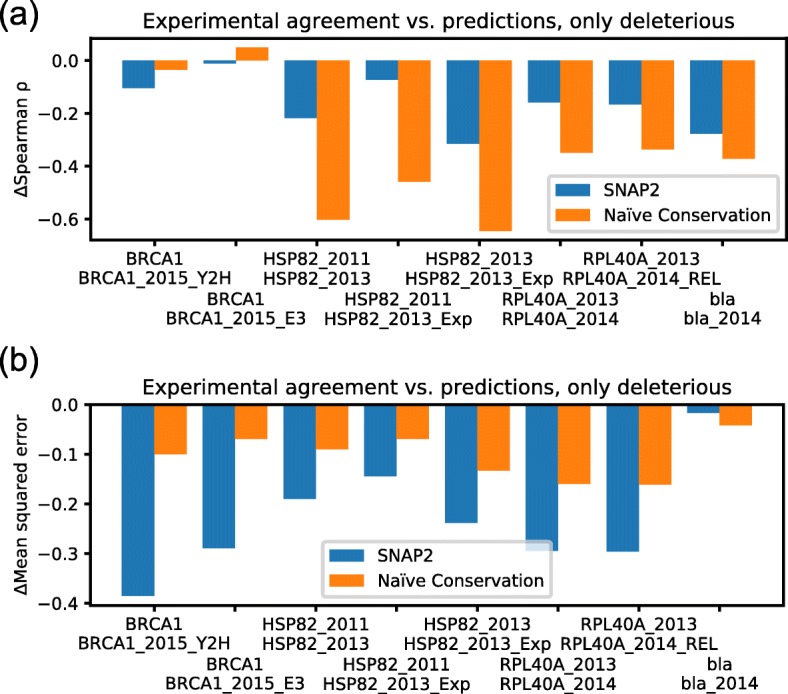
Experimental agreement vs. predictions. For every pair of experimental measurements on the same protein (Table S1), the agreement between two experiments and that between each experiment and the predictions of SNAP2 and Naïve Conservation are compared. **a** ∆ρ = 0.5*(ρ(× 1,p1) + ρ(× 2,p2)) - ρ(× 1,× 2), (b) ∆MSE = MSE(× 1,× 2) - 0.5*(MSE(× 1,p1) + MSE(× 2,p2)). Where × 1/× 2 are the experiments and p1/p2 the predictions on the two experiments, all of which are calculated based on the largest possible set of SAVs. Negative values on the y-axes thus imply that the agreement between experiments is higher than that between experiment and prediction, positive values that predictions agree more

The above comparisons of experimental and predicted SAV effects raise the question of what agreement can realistically be obtained. One proxy for an answer is the comparison of different DMS studies conducted on the same protein. Such data were available for 11 measurements on 4 proteins (Table S8, Fig. S7); unfortunately, Envision predictions were available for only one of those proteins (BRCA1). For deleterious SAVs, the lowest correlation was that between two measurements on breast cancer type 1 susceptibility protein, BRCA1 and BRCA1_2015_E3 (ρ = 0.21, Fig. S7b). Rather than experimental noise, the low correlation might also originate from different experimental setups employed for multi-functional proteins such as BRCA1. The strong correlation (ρ = 0.93) between two experiments that measured the same condition for bla (beta-lactamase TEM precursor; bla and bla_2014, Fig. S7h) provided a single case in strong support of such an explanation. To compare prediction methods and experiments, we assessed the difference in ρ and MSE for each combination of the 11 measurements (Fig. 3). Experiments clearly agreed more with each other than with SNAP2 and Naïve Conservation on the same datasets (Fig. 3: all values negative).

Experiments did not correlate at all with each other for beneficial effect (mean ρ = 0.03) although the MSE remained low (mean MSE = 0.05, Table S8, Fig. S8). The major issue for this comparison was the small number of only 572 SAVs.

### Assessment of binary classification (neutral/effect) similar to regression

Scores from binary classification methods (neutral or effect) are often assessed through receiver operating characteristic (ROC) curves avoiding to choose particular thresholds to distinguish neutral and effect. Toward this end, we assigned classes to SAVs through normalization by experimental measurements of synonymous variants [60] (Methods). Other solutions are feasible, each with their own ad hoc parameter choices and flaws implying that the following results provide one snapshot instead of a sustained method ranking.

**Fig. 4:**
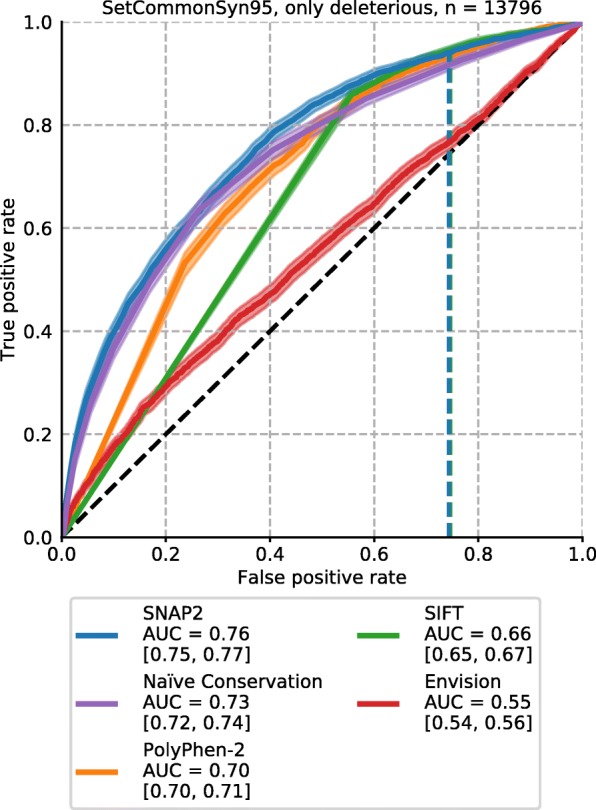
Classification performance of all prediction methods. Shown are ROC curves for 13,796 deleterious effect SAVs which were classified into either neutral, defined by the middle 95% of the scores from synonymous variants, or effect (*SetCommonSyn95*). Shaded areas around lines denote 95% confidence intervals. The legend denotes the AUC for each of the five prediction methods, along with the 95% confidence intervals. Horizontal dashed lines denote the default score threshold used by SNAP2 (blue) and SIFT (green)

On the 3209 deleterious effect SAVs of *SetCommonSyn95* (10,587 neutral, Table 1, Fig. S9), SNAP2 achieved the highest area under the curve (AUC, 0.76, 95% CI [0.75, 0.77]). It was the only method statistically significantly better than Naïve Conservation (0.73 [0.72, 0.74], Figs. 4, S10 Table S9). Precision-recall curves also highlighted the smooth transition of SNAP2 scores opposed to those for Naïve Conservation although the peak performance was similar for both (Fig. S11). Envision - not developed for this task - performed better than random, but clearly worse than the classification methods (AUC = 0.55 [0.54, 0.56]). However, the four proteins considered here (BRCA1, PPARG, PTEN and TPMT), also correlated above average for SNAP2, PolyPhen-2 and SIFT (Table S3). Using different thresholds in severity to classify SAVs did not qualitatively change these major findings (*SetCommonSyn90*, *SetCommonSyn99*, Fig. S12a-b).

At their default thresholds SIFT, PolyPhen-2, and SNAP2 consider over two thirds of the neutral variants to have an effect. Interestingly, the behavior of Envision trained on DMS data was the reverse as previously illustrated by the maximal scores reaching only up to 61% of the possible maximal values (and thereby contributing to a seemingly low MSE).

Beneficial SAVs were also difficult to classify: PolyPhen-2 and SNAP2 performed best with AUC = 0.62, followed by SIFT, while Envision predictions were not better than random (Fig. S13, Fig. S12c-d, Table S9). Naïve Conservation also performed significantly worse at a level of random predictions.

## Discussion

### No clear winner in predicting effect variants

We compared the predictions of five methods with SAV effects determined by DMS experiments. SNAP2 was trained on binary classification data (effect or neutral). Nevertheless, predictions have been shown to correlate with effect strength [5, 66, 67]. To a degree, the Deep Mutational Scanning (DMS) data replicated this finding, highlighting that even methods trained for classification capture aspects of effect strength.

Sorting DMS scores into 20 bins and including classification methods SIFT and PolyPhen-2 in the analysis, all methods indicated better recognition of high effect SAVs. This finding might be attributed to the bias of classifications methods towards high effect variants, a common criticism in the field [68–71]. We observed the same trend for Naïve Conservation exclusively using PSI-BLAST profiles to predict SAV effects. This emphasized the importance of this signal but to some extend also explained the traditional classification methods’ bias since they all rely on this input.

The significantly better performance of Envision in estimating the precise degree of effect especially suggested value in this approach. However, the low MSE was largely explained by that Envision correctly predicted the overall distribution of experimental scores. Thus, the definite distinction between ‘good prediction’ and ‘advantageous bias’ remained elusive.

When treating DMS effect scores as binary assignments (neutral or effect), ROC curves highlighted the high false positive rates of the evaluated classification methods. A similar perspective on over-prediction has recently been observed for ClinVar data [69]. Over-prediction might be encouraged by the way many users of prediction methods mistakenly chose their tools, namely by testing a small set of SAVs they know have an effect and valuing methods highest when they predict effects for more of those.

### Family conservation carries most important signal

Most surprising was the overall good performance of Naïve Conservation. Disease causing SAVs from OMIM typically affect the most conserved residues [46], and machine-learning based predictions have been criticized to largely capture conservation [17, 70, 72–74]. Furthermore, simple conservation patterns can capture aspects of variant effects [75]. Our findings partially validated this for DMS experiments, although the effect distributions observed by DMS and predicted by Naïve Conservation differed substantially (Fig. 1c, gray distributions). Another recent analysis also found a method heavily relying on evolutionary information as one of the best performers on DMS data, although more sophisticated than our naïve approach [48, 76].

### Beneficial effects neither correctly predicted, nor consistent between experiments

The bad correlation and classification performance of beneficial effect SAVs by all methods suggested those to have distinctly different signatures than deleterious SAVs, missed by current approaches. Generally, SAVs with neutral or beneficial effects are often not recognized well [69, 77]. In part, this is attributable to the lack of respective experimentally verified data useable for training sets. For beneficial effect variants, the rise of DMS studies could help to alleviate this problem and lead to the development of less biased methods.

Agreement between experimental studies was particularly low for beneficial effect SAVs. Maybe DMS assays are still biased towards measuring deleterious effects. These results put the seemingly poor predictions of beneficial SAVs into perspective. Generally, the wide variation of correlation between experiments for different datasets/proteins has also been observed in another recent DMS analysis [48].

## Conclusions

Deep mutational scanning (DMS) studies set out to explore the relation between protein sequence and molecular function. We collected 22 DMS experiments and focused on single amino acid variants (SAVs, also referred to as missense mutations or non-synonymous SNVs). Most studies probe only a small subset of all possible variants (for a protein with N residues, there are 19*N non-native SAVs). Two experiments probing the same protein tended to agree more with each other than with predictions for deleterious effect (Fig. 3). Nonetheless, experiments also disagreed significantly (Table S8). No single measure captured all aspects of the comparison between experiments and predictions, e.g. the ranking of methods changed crucially depending on the measure used to compare (Table 2, SOM_Note2).

We analyzed five variant effect prediction methods: *Envision* was trained on DMS data, *PolyPhen-2*, *SIFT* and *SNAP2* were methods developed to classify into effect/neutral, and *Naïve Conservation* (essentially using PSI-Blast conservation to predict effect/neutral) was added to gauge the importance of evolutionary conservation for the prediction. For deleterious SAVs, all methods reached slightly positive Spearman ρ correlations with the DMS experiments (Fig. 1). The classification method SNAP2 correlated most with effect strength, although most of the correlation was explained by simple conservation. The lowest mean squared error (MSE) was achieved by Envision. Its MSE was as low as that between experiments, although most of the low MSE could be explained by correctly predicting the distribution of scores (Fig. 1, Fig. S2a). All methods performed better on SAVs with deleterious (akin to loss-of-function) than with beneficial (gain-of-function) effect. However, experimental agreement was also almost non-existing for beneficial effects.

Although binary classification methods, surprisingly, captured aspects of non-binary measurements, they performed much better for the binary classification task (projecting DMS results onto neutral vs. effect; Fig. 4). Notably, Naïve Conservation captured effect better than some more advanced tools. Methods performed better for SAVs with stronger experimental effect scores (Fig. 2: higher toward right), although most classifiers tended to substantially over-predict at their default scores (Fig. 4). Overall, our analyses confirm some of the trends from other reviews of DMS data [48, 49].

The challenge for the next generation of prediction methods will be to learn from the diversity of DMS. To give just one example: OMIM, a popular source of training data, contained ~ 11,000 SAVs referenced in dbSNP (02/2019, [78]). This is a magnitude matched by a single large DMS experiment. The generality of a single SAV might not be comparable between the sets, yet DMS opens up variant effect prediction to new methodologies, possibly even to deep learning approaches [79, 80]. The enriched data might also allow methods to distinguish between toggle and rheostat positions [73]. Furthermore, DMS studies contain many beneficial effect SAVs that have, so far, been underrepresented. Finally, DMS focuses on molecular function, i.e. some of the disruptive SAVs (deleterious or beneficial) might correspond to clinically benign SAVs. Nevertheless, DMS will likely give rise to new methods better predicting SAV effects upon molecular protein function and upon organisms. In fact, growth-based DMS assays have been shown to be predictive of human disease SAVs in a recent study [48]. Therefore, a combination of experimental data with new prediction methods might be what is needed to attain the goals of precision medicine.

## Methods

### Dataset collection

**Fig. 5:**
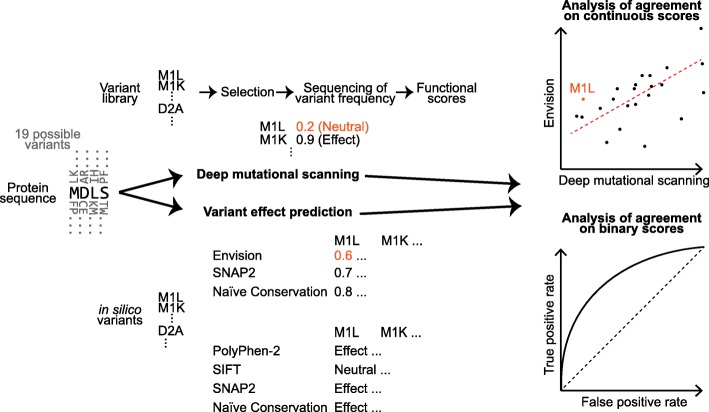
Concept of analysis. Experimental scores of variant effects (missense mutations, or single amino acid variants, labelled SAVs) from Deep Mutational Scanning (DMS) experiments were compared to in silico prediction methods. *Envision* was the only method developed on DMS data; it provides continuous scores mirroring the DMS data. SIFT, PolyPhen-2 can be evaluated as binary classification methods. SNAP2 is a classification method but provides continuous scores that can also be used. Naïve Conservation is provided as a baseline for both cases

Figure 5 sketches the basic workflow of this analysis. We retrieved all DMS datasets available by June 2019 that report over 100 SAVs available from the literature. Functional effect scores were taken directly from the supplemental material published or requested from the authors (Table S10). The data were formatted in a variety of formats including Excel, and tab- or comma-separated files. Scores were manually mapped either to the UniProtKB identifier given in the publication or to its closest BLAST match (Table S11) [44, 81]. Six of the 22 experiments contained up to five substitutions (pairwise sequence identity ≥98%); those were maintained for prediction. We refer to the combined data as *SetAll* (66,089 SAVs) supplemented by *SetCommon* with 32,981 SAVs for which we had a prediction from every method tested (Table 1). *SetCommon* contained SAVs from ten of the 22 experiments: YAP1, MAPK1, BRCA1, CCR5, CXCR4, GAL4, PPARG, PTEN, TPMT, and Ube4b (Table S1). During completion of this manuscript, MaveDB, a centralized resource of multiplexed assays of variant effect has been published [8, 82]. MaveDB identifiers exist for ten of our 22 datasets (November 2019, Table S10).

*SetAll* contained several proteins with multiple independent experimental measurements. Inclusion of additional sets analyzed previously [49], yielded a total of three measurements for Hsp82 and BRCA1 and two for both beta-lactamase and ubiquitin (Table S1) [27, 28, 83]. Performance measures were calculated only on SAVs and not between DMS measurements from the same publication. For analysis of beneficial effect SAVs, all studies on Hsp82 had to be excluded since the sets contain only three of those SAVs each.

### Processing functional effect scores

Several DMS studies provide multiple effect scores for the same protein of which we decided on only one per set (Table S12). In the following processing, effect scores were left as provided by the authors as much as possible but adjusted such that the wild-type score for each measurement (Table S13) became 0, and larger values denoted more effect. Next, scores were interpolated, separately for each of the 22 DMS measurements, to lie between 0 and 1 (highest effect). This interpolation did not affect Spearman ρ or the mean squared error within each dataset. Beneficial and deleterious effects had to be analyzed separately because experimental assays were not symmetrical and further normalization might over- or underrepresent effects. The resulting score distributions differed significantly between experiments (e.g. in contrast to the more homogeneous subset used previously [49]).

We also created sets with binary classifications (effect vs. neutral) from all DMS studies with synonymous variants: The middle 95% of effect score values from synonymous variants was used to define which SAVs were considered neutral. All SAVs outside this range were considered as effect. We applied the same procedure using 90% or 99% of synonymous variants’ values and refer to the thresholding schemes as *syn90*, *syn95*, and *syn99*. Applying these schemes to the four experiments in *SetCommon* which have synonymous variants (BRCA1, PTEN, TPMT, PPARG) yields *SetCommonSyn90|95|99*. Again, deleterious and beneficial effect SAVs were analyzed separately.

### Performance measures

Experiments and predictions were compared through three measures (SOM_Note3, SOM_Note2): (1) **mean squared error** (**MSE**) calculated with the scikit-learn metrics module [84]; (2) **Pearson R** (pearsonr) and (3) **Spearman ρ** (spearmanr) both calculated with the SciPy stats module [85]. For convenience linear least-squares regression lines (linregress) were added to the correlation plots. Pearson R was added for ease of comparison to others but not discussed as it is not robust and most datasets violated both its validity assumptions (normal distribution & absence of significant outliers [86]). We further found no evidence to supplement MSE by a measure more robust to outliers (SOM_Note2). 95% confidence intervals (**CIs**) for R, ρ and MSE were estimated using a percentile bootstrap with 1000 random samples with replacement.

The performance of binary predictions (effect vs. neutral) was measured through receiver operating characteristic (**ROC**) curves and the area under those curves (**AUC**) calculated through the pROC package in R, which was also used to calculate 95% confidence intervals of ROC (ci.se) and AUC (ci.auc) [87, 88]. Additionally, precision-recall curves were created using scikit-learn (precision-recall-curve). These are defined with TP as true positives (predicted and observed as effect), FP as false positives (predicted as effect, observed as neutral), and FN as false negatives (predicted neutral, observed effect): Precision = TP/(TP + FP), Recall = True Positive Rate = TP/(TP + FN) and False Positive Rate = FP/(FP + TN).

### Prediction methods

The sequences determined during dataset collection were used as input to a set of commonly used variant effect prediction methods. Each method was run to predict the effect of all 19 non-native amino acids at every position in the protein. *SNAP2* [38] was run locally using default parameters on UniProtKB (Release 2018_09). *SIFT* version 6.2.1 [39] was run locally (UniProtKB/TrEMBL Release 2018_10). *PolyPhen-2* [37] predictions were retrieved from the webserver in batch mode with classification model humdiv on genome assembly GRCh37/hg19 and default parameters [89]. Predictions failed for all relevant residues of the three DMS studies on Hsp82. *Envision* [49] predictions were retrieved online which requires UniProtKB identifiers as input [90]. Therefore, Envision predictions could be analyzed only for ten proteins (Table S14). While SNAP2 and SIFT predicted all SAVs, PolyPhen-2 and Envision failed for some residues, shrinking the size of the datasets. We always report performance on the largest common subset of SAVs per dataset.

As a baseline, predictions were also created by running PSI-BLAST with three iterations on UniProtKB (Release 2018_09). Scores from the resulting profile (position-specific scoring matrix) had their signs flipped and were then directly used as a measure of effect, i.e. less frequent substitutions have a higher effect than conserved ones. We refer to this method as Naïve *Conservation*. The prediction was not intended to be the most accurate conservation score possible but rather to represent a suitable baseline since (PSI-)BLAST results are used in some way as input feature by all methods analyzed here.

For SIFT, scores were reversed such that higher values implied higher effect. The same was done for Envision predictions of deleterious effect. Envision predictions of beneficial effect were treated separately and mapped to the range of [0,0.2]. This yielded the same performance than scaling between [0,1] or no scaling (SOM_Note4). Finally, prediction scores of all methods were adjusted to lie between 0 (no effect) and 1 (highest effect) using the theoretical maximum and minimum prediction value of every method.

## Supplementary information

**Additional file 1.** Supporting Online Material (SOM) containing additional figures, tables and notes.

## Data Availability

The datasets generated and code for their analysis are available on Mendeley Data (10.17632/2rwrkp7mfk.1).

## Abbreviations

AUC: Area under the ROC curve
CI: Confidence interval
DMS: Deep mutational scanning
MSE: Mean squared error
ROC: Receiver operating characteristic
SAV: Single amino acid variant
